# Implementation and scale-up of physical activity and behavioural nutrition interventions: an evaluation roadmap

**DOI:** 10.1186/s12966-019-0868-4

**Published:** 2019-11-07

**Authors:** Heather McKay, Patti-Jean Naylor, Erica Lau, Samantha M. Gray, Luke Wolfenden, Andrew Milat, Adrian Bauman, Douglas Race, Lindsay Nettlefold, Joanie Sims-Gould

**Affiliations:** 1 Centre for Hip Health and Mobility, Vancouver Coastal Health Research Centre, 7th Floor Robert H.N. Ho Research Centre, 795-2635 Laurel St, Vancouver, BC V5Z 1M9 Canada; 2 Department of Family Practice, University of British Columbia, 3rd Floor David Strangway Building, 5950 University Boulevard, Vancouver, BC V6T 1Z3 Canada; 3 School of Exercise Science, Physical Health and Education, Faculty of Education, University of Victoria, PO Box 3015 STN CSC, Victoria, BC V8W 3P1 Canada; 4 School of Medicine and Public Health, University of Newcastle, Callaghan, New South Wales 2308 Australia; 5 Hunter New England Population Health, Wallsend, New South Wales 2287 Australia; 6 The New South Wales Ministry of Health, North Sydney, New South Wales 2059 Australia; 7 Sydney School of Public Health, University of Sydney, Charles Perkins Centre, Building D17, Sydney, New South Wales 2006 Australia

**Keywords:** Implementation science, Exercise, Healthy eating, Scalability, Dissemination, Public health

## Abstract

**Background:**

Interventions that work must be effectively delivered at scale to achieve population level benefits. Researchers must choose among a vast array of implementation frameworks (> 60) that guide design and evaluation of implementation and scale-up processes. Therefore, we sought to recommend conceptual frameworks that can be used to design, inform, and evaluate implementation of physical activity (PA) and nutrition interventions at different stages of the program life cycle. We also sought to recommend a minimum data set of implementation outcome and determinant variables (indicators) as well as measures and tools deemed most relevant for PA and nutrition researchers.

**Methods:**

We adopted a five-round modified Delphi methodology. For rounds 1, 2, and 3 we administered online surveys to PA and nutrition implementation scientists to generate a rank order list of most commonly used; i) implementation and scale-up frameworks, ii) implementation indicators, and iii) implementation and scale-up measures and tools. Measures and tools were excluded after round 2 as input from participants was very limited. For rounds 4 and 5, we conducted two in-person meetings with an expert group to create a shortlist of implementation and scale-up frameworks, identify a minimum data set of indicators and to discuss application and relevance of frameworks and indicators to the field of PA and nutrition.

**Results:**

The two most commonly referenced implementation frameworks were the Framework for Effective Implementation and the Consolidated Framework for Implementation Research. We provide the 25 most highly ranked implementation indicators reported by those who participated in rounds 1–3 of the survey. From these, the expert group created a recommended *minimum data set* of implementation determinants (*n* = 10) and implementation outcomes (*n* = 5) and reconciled differences in commonly used terms and definitions.

**Conclusions:**

Researchers are confronted with myriad options when conducting implementation and scale-up evaluations. Thus, we identified and prioritized a list of frameworks and a minimum data set of indicators that have potential to improve the quality and consistency of evaluating implementation and scale-up of PA and nutrition interventions. Advancing our science is predicated upon increased efforts to develop a common ‘language’ and adaptable measures and tools.

## Background

Interventions that work, must be effectively delivered at scale to achieve health benefits at the population level [1]. Despite the importance of scaling-up health promotion strategies for public health, only 20% of public health studies examined ways to integrate efficacious interventions into real-world settings [2]. Within health promotion studies, only 3% of physical activity (PA) [3] and relatively few behavioural nutrition (nutrition) interventions were implemented at large scale [4]. Implementation is the process to integrate an intervention into practice within a particular setting [5]. Scale-up is “the process by which health interventions shown to be efficacious on a small scale and or under controlled conditions are expanded under real world conditions into broader policy or practice” [1].

The concept of implementation and scale-up are closely inter-twined—there is not always a clear delineation between them. From our perspective, and others [6, 7], implementation and scale-up co-exist across a continuum or ‘program life-cycle’ that spans development, implementation, maintenance and dissemination (in this paper we use ‘dissemination’ interchangeably with ‘scale-up’). In an ideal world, only interventions that demonstrated efficacy in purposely designed studies would be scaled-up. In reality the boundary between implementation and scale-up is less clear, as the scale-up process is often non-linear and phased [8]. Further, theoretical frameworks and indicators that guide implementation and scale-up processes are often similar [9].

More than 60 conceptual frameworks [10], more than 70 evidence-based strategies [11], and hundreds of indicators were developed [9] to guide implementation and scale-up of health interventions. PA or nutrition researchers and practitioners may find it challenging to navigate through this maze to design, implement, and evaluate their interventions [12]. We define implementation frameworks (including theories and models) as principles or systems that consist of concepts to guide the process of translating research into practice and describe factors that influence implementation [10]. Implementation strategies are methods used to enhance adoption, implementation, and sustainability of an intervention [13]. Scale-up frameworks focus on guiding design of processes and factors that support uptake and use of health interventions shown to be efficacious on a small scale and or under controlled conditions to real world conditions.

**Fig. 1:**
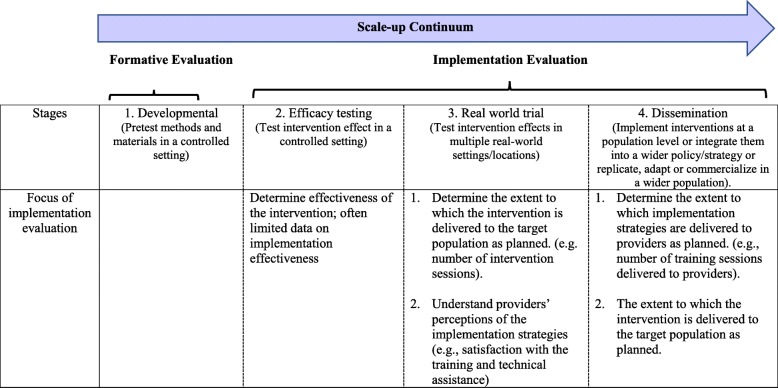
The focus of implementation evaluation along the scale-up continuum

Along the continuum from efficacy to scale-up studies (Fig. 1) [3], the focus of implementation evaluation shifts. For efficacy and effectiveness studies, implementation evaluation centres on how well the intervention was delivered to impact selected health outcomes of a target population. As scale-up proceeds, the focus more so is to evaluate specific implementation strategies that support uptake of the intervention on a broad scale [14]. Evaluation specifies implementation indicators relevant to delivery of the intervention *and* delivery of implementation strategies. We define *implementation indicators* as specific, observable, and measurable characteristics that show the progress of implementation and scale-up [15]. They comprise two categories: *implementation outcomes* refer to the effects of deliberate actions to implement and scale-up an intervention [16]. *Implementation determinants* refer to the range of contextual factors that influence implementation and scale-up [17].

Thus, the impetus for our study is threefold. First, we respond to a need voiced by colleagues conducting nutrition and PA intervention studies, to provide a simplified pathway to evaluate the implementation of interventions across a program life cycle. Second, nutrition and PA interventions that were scaled-up are beset with differences in terminology, often lack reference to appropriate frameworks and assess few if any, implementation and scale-up indicators [18, 19]. We sought to enhance clarity on these issues. Finally, there are few valid and reliable measures and tools – and sometimes none at all – for evaluating implementation and scale-up processes [20]. Thus, it is difficult to interpret or compare results across studies [21], slowing the progression of our field. Ultimately, we aim to extend discussions and alleviate barriers to conducting much needed implementation and scale-up studies in PA and nutrition.

Specifically, with a focus on PA and nutrition, we sought to identify frameworks that can be used to design and evaluate implementation and scale-up studies and common implementation indicators (as a “minimum data set”) that have relevance for researchers. We acknowledge the vital role of implementation strategies. However, for our study we do not describe or discuss specific implementation strategies, as these have been comprehensively reviewed elsewhere [11, 13]. Therefore, we adopted a modified Delphi methodology with an international group of implementation scientists in PA and nutrition to address three key objectives; 1. to identify and describe most commonly used frameworks that support implementation and scale-up, 2. to identify and define preferred indicators used to evaluate implementation and scale-up, and 3. to identify preferred measures and tools used to assess implementation and scale-up.

## Methods

### Research design

We adopted a five-round modified Delphi methodology [22–24]. For rounds 1, 2, and 3 we administered online surveys to PA and nutrition implementation and scale-up scientists to generate a rank order list of most commonly used frameworks, indicators, and measures and tools. For rounds 4 and 5, we conducted in-person meetings with an expert group to better understand the application and relevance of responses that emerged in rounds 1, 2, and 3. The goal of the expert group was to reach consensus on a shortlist of frameworks and a minimum data set of implementation indicators for implementation and scale-up studies in PA and nutrition. The Institutional Review Board at the University of British Columbia approved all study procedures (H17–02972).

### Participants

We used a snowball sampling approach to recruit participants for our Delphi survey. First, we identified potential participants from our professional connection with the International Society of Behavioural Nutrition and PA (ISBNPA), Implementation and Scalability Special Interest Group (SIG). The SIG aims to provide a platform to facilitate discussion and promote implementation science in the field of PA and nutrition. The international group of SIG early and mid-career investigators, senior scientists, practitioners and policy makers have a common interest in evaluating implementation and scale-up of PA and nutrition interventions.

Second, we contacted the list of SIG attendees who agreed to be contacted to participate in research relevant to SIG interests (*n* = 18), all of whom attended the ISBNPA SIG (2017) meeting. All researchers or practitioners engaged in nutrition, PA or sedentary behaviour research, who had published at least one paper related to implementation or scale-up, were eligible to participate.

Third, we supplemented the recruitment list using a snowball sampling approach with input from an Expert Advisory group. The Expert Advisory group (*n* = 5) was advisory to the SIG and had > 10 years of experience conducting PA and/or nutrition implementation or scale-up studies. They identified 14 other eligible participants. Our final recruitment sample comprised 32 eligible implementation or scale-up science researchers and practitioners. Of these, 19 participants (79%; 13 women) completed the first round, 11 (48%, 9 women) completed round 2, and 16 (70%, 11 women) completed round 3. Participants had one to ten (*n* = 13), 11 to 20 (*n* = 3), or more than 20 (*n* = 3) years of experience in implementation science. Most participants were university professors, two were practitioners/decision makers, and one was a postdoctoral researcher.

We established an expert group comprised of 11 established scientists (eight university professors, two researcher/policy makers, cross appointed in academic and government public health agencies, and one postdoctoral researcher) from different geographical regions (North America = 6, Australia = 4, Netherlands = 1). Their research in health promotion and public health spanned implementation and scale-up of nutrition or PA interventions across the life span. They had expertise in design and/or evaluation of implementation indicators, and measures and tools. All expert group members participated in round 4. In round 5, a subset of the most senior researchers (*n* = 5; > 10 years of experience in implementation and scale-up science) engaged in a pragmatic (based on availability), intensive face-to-face meeting to address questions and discrepancies that surfaced during the Delphi process.

### Data collection and analysis

#### Round 1 survey: open

The aim was to develop a comprehensive list of frameworks, indicators, and measures and tools most commonly used in implementation and scale-up of PA and nutrition interventions. We invited participants to complete a three-section online survey (FluidSurvey; Fluidware Inc., Ottawa, ON, Canada); section 1. participants provided demographic data (e.g., age, gender, number of years conducting implementation and/or scale-up science research); section 2. we provided a list of implementation frameworks, indicators, and measures and tools generated by attendees during a SIG workshop. Survey participants were asked to include or exclude items as relevant to implementation science (based on their knowledge and experience), and to also note if items were misclassified. Participants were also asked to suggest other ‘missing’ implementation frameworks, indicators, or measures and tools they deemed relevant to PA and nutrition in implementation science; section 3. participants replicated the process above with a focus on scale-up science (see Additional file 1 for the full survey).

*Analysis:* We retained items that received ≥70% support from participants [22]. We excluded items that received > 70% support but were specific to individual-level health behaviour change. We reclassified some items as per participant responses. For example, socio-ecological and transtheoretical models were considered behaviour change theories, not implementation or scale-up frameworks. RE-AIM was reclassified as an evaluation, rather than an implementation framework [25]. We used categories as per Nilsen [10] to classify data into process models, determinant frameworks, classic theories, implementation theories, and evaluation frameworks. As this classification system did not differentiate between implementation and scale-up frameworks, we added a scale-up frameworks category. We aligned indicators with definitions in the published implementation science literature [16, 17, 26, 27] and implementation science websites [e.g., WHO; SIRC; Expand Net; Grid-Enabled Measures Database]. As participants did not clearly differentiate between implementation and scale-up indicators, we collapsed indicators for round 2 as many applied to implementation evaluation across the program life cycle [7]. Results from round 1 were compiled into an interactive spreadsheet and used as a survey for round 2.

#### Round 2: selecting and limiting

The purpose was to differentiate among and summarize participant responses. Responses included implementation and scale-up frameworks, theories, models, indicators, and measures and tools. Ultimately we wished to create a shortlist of items from round 1, that were most commonly used in implementation and scale-up of PA and nutrition interventions. To do so we emailed participants an interactive spreadsheet comprised of three sections; section 1. implementation frameworks and models (*n* = 28), section 2. scale-up frameworks and models (*n* = 16), section 3. implementation indicators (with definitions) (*n* = 106) and measures and tools (*n* = 15). Each section included items retained in round 1 and new items added by participants during that round. Within each section, items were listed alphabetically. Participants were asked to: i) denote with a check whether items were: “relevant – frequently used”; “relevant – sometimes used”; “relevant – do not use”; “not relevant”; “don’t know”; ii) denote with an asterisk the top five most relevant frameworks; and iii) describe factors that influenced their choices [12] (Additional file 2).

Any reference added by a participant in round 1 was provided to all participants in round 2. Participants selected “don’t know” if they were unfamiliar with an item in the survey.

*Analysis:* After round 2, we use the term *frameworks* to represent theories and conceptual frameworks [9] and added the term *process models* to refer specifically to both implementation and scale-up process guides. We operationalize implementation frameworks as per our definition in Background [10]. We differentiate these from scale-up frameworks that guide the design of scale-up processes to expand health interventions shown to be efficacious on a small scale and or under controlled conditions to real world conditions. Some implementation frameworks are also relevant for and can be applied to scale-up. We ranked frameworks, process models, indicators, and measures and tools based on the frequency of checklist responses (%). Finally, as input from participants about preferred measures and tools was very limited, we excluded this aspect of the study from subsequent rounds.

#### Round 3: ranking

The purpose was to create a rank order list of most frequently used frameworks, process models and indicators for implementation and scale-up of PA and nutrition interventions. For round 3 the spreadsheet consisted of three sections [10]: section 1. top five implementation frameworks and process models; section 2. top five scale-up frameworks and process models; and section 3. top 25 implementation indicators. Rank order was based on preferred/most frequently used items noted in round 2. Participants were asked to rank items and comment as per round 2.

*Analysis:* We sorted and ranked implementation frameworks, scale-up frameworks, and process models based on checklist responses (%). We ranked 25 indicators most relevant to and frequently assessed by participants. When indicator rankings were the same, we collapsed indicators into one category based on rank score (e.g., 11–15; 20–25).

#### Rounds 4 and 5: expert review

For Round 4, the expert group convened for eight-hours. The purpose of the meeting was to discuss frameworks, process models, and indicators related to implementation and scale-up of PA and nutrition interventions. Activities spanned presentations and interactive group discussions. For one exercise, the expert group was provided a shortlist of frameworks, process models, and indicators generated in the round 3 survey. They were asked to place frameworks, process models, and indicators in rank order, from most to least relevant among those most often used in their sector. We define sector as an area of expertise or services in health care or health promotion that is distinct from others. Research assistants collected field notes during all sessions.

*Analysis:* We ranked frameworks and process models and implementation indicators based on expert group feedback. Research assistants summarized meeting notes to capture key issues and to guide data interpretation.

Round 5 comprised two 4-h in person discussions with a subset of senior scientists from the expert group. The purpose was to: 1. reach consensus on frameworks and process models most relevant to PA and nutrition researchers who wished to conduct implementation and scale-up studies, 2. identify a core set of implementation indicators for assessing implementation and scale-up of PA and nutrition interventions, 3. within implementation indicators, differentiate implementation outcomes from implementation determinants, 4. agree upon common names and definitions for indicators that apply to implementation or scale-up science.

*Analysis:* The expert group was provided a large spreadsheet that listed frameworks, process models, and indicators generated from round 4. We defined indicators based on the published implementation science literature [16, 17, 26, 27] or implementation science websites [e.g., WHO; SIRC; Expand Net; Grid-Enabled Measures Database].

For some indicators we found more than one definition. However, they most often described similar concepts. To illustrate, the definition of compatibility contained the terms appropriateness, fit and relevance [28]. The dictionary definition of appropriateness, contains the terms fit and suitability. When this occurred the expert group agreed upon one definition. When different terms were used to represent similar indicators, the expert group selected one term to refer to the indicator (e.g., compatibility over appropriateness). Meeting notes from in-person meetings were summarized narratively to inform results and identify critical issues.

## Results

### Frameworks and process models

The two most commonly referenced implementation frameworks were the Framework for Effective Implementation [17] and the Consolidated Framework for Implementation Research (CFIR) (Table 1) [27]. Both frameworks can be used to guide scale-up evaluation. Scale-up frameworks that participants identified (Table 1) were more appropriately reclassified as process models. For completeness, we acknowledged the importance of Diffusion of Innovation Theory [37] and a broad reaching conceptual model [26] as they were often noted by participants. We classify them as comprehensive theories or conceptual models within the scale-up designation (Table 1).

**Table 1:** Implementation and scale-up frameworks and process models that surfaced most often

Implementation	
*Frameworks* 1. Framework for Effective Implementation [17] 2. Consolidated Framework for Implementation Research (CFIR) [27] 3. Dynamic Sustainability Framework [29]	
Scale-Up	
*Frameworks* 1. Scaling Up Health Service Innovations - A Framework for Action [30] 2. **Interactive Systems Framework for Dissemination andImplementation** [31] 3. **Scaling-Up: A Framework for Success** [32]	
*Process Models* 1. Steps to Developing a Scale-Up Strategy [33] 2. Review of Scale-Up/Framework for Scaling Up Physical Activity Interventions [34] 3. A Guide to Scaling Up Population Health Interventions [35, 36]	
*Comprehensive Theories or Conceptual Models* 1. Diffusion of Innovations [37] 2. Conceptual Model for the Spread and Sustainability of Innovations in Service Delivery and Organization [26]	

*Note:* Additional resources recommended by experts who participated in rounds 4 and 5 of the modified Delphi process are bolded

### Implementation determinants and outcomes

We provide the 25 most highly ranked indicators from rounds 1–4 (Table 2). If we were unable to differentiate among single rank scores, we collapsed indicators into one rank group. To illustrate, adherence, appropriateness, cost, effectiveness, and fidelity were ranked the same by participants so they were grouped together in an 11–15 category.

**Table 2:** The 25 most highly ranked indicators reported by those who participated in Delphi Rounds 1–4

Indicators	Ranking	
	Round 1–3	Round 4
Acceptability	1	4–11
Adoption	2	3
Adaptability/adaptation	3	25
Barriers	4	19–22
Context	5	4–11
Implementation	6	4–11
Feasibility	7	12–16
Dose delivered (completeness)	8	17–18
Reach	9	1
Dose received (exposure)	10	4–11
Adherence	11–15	12–16
Appropriateness	11–15	23–24
Cost	11–15	2
Effectiveness	11–15	4–11
Fidelity	11–15	4–11
Culture	16–20	12–16
Dose (satisfaction)	16–20	19–22
Maintenance	16–20	19–22
Recruitment	16–20	19–22
Sustainability	16–20	4–11
Complexity	21–25	23–24
Dose	21–25	12–16
Efficacy (of interventions)	21–25	12–16
Innovation characteristics	21–25	23–24
Self-efficacy	21–25	17–18

Table 3 provides the *minimum data set* generated by the expert group in rounds 4 and 5. The first column described the name of the recommended implementation indicators, separated by implementation outcomes (*n* = 5) and determinants (*n* = 10). We minimized the data set by; 1. excluding terms that were generic measures rather than specific indicators (i.e., barriers, facilitators, implementation, recruitment, efficacy and effectiveness); 2. choosing one name for indicators with different names but with similar definitions. (i.e., fidelity over adherence, sustainability over maintenance, dose delivered over dose and compatibility over appropriateness); 3. selecting one definition for determinants and outcomes. Preferred terms were selected during in person discussions among the expert group. Reasons for experts’ preference included terms most commonly used and ‘understood’ in the health promotion literature and the public health sector, and terms most familiar to practitioners and other stakeholder groups (e.g. government).

**Table 3:** A minimum data set of implementation outcomes and determinants

Implementation outcomes	Delivery of the intervention	Delivery of implementation strategies
	Definitions	Definitions
1. Adoption	Proportion and representativeness of providers or the delivery team* that deliver an intervention [25].	Proportion and representativeness of the support system* that utilize implementation strategies.
2. Dose delivered (dose)	Intended units of each intervention component delivered to participants by the delivery team [38].	Intended units of each implementation strategy delivered by the support system.
3. Reach	Proportion of the intended priority audience (i.e., participants) who participate in the intervention [39].	Proportion of the intended priority populations (organizations and/or participants) that participate in the intervention.
4. Fidelity (adherence)	The extent to which an intervention is implemented as it was prescribed in the intervention protocol - by the delivery team [5].	The extent to which implementation strategies are implemented as prescribed in the scale-up plan - by the support system.
5. Sustainability (maintenance)	Whether an intervention continues to be delivered and/or individual behaviour change is maintained; intervention and individual behaviour change may evolve or adapt with continued benefits for individuals after a defined period of time [40].	Whether implementation strategies continue to be delivered and/or behaviour change at the system level are maintained; implementation strategies and behaviour change at the system level may evolve or adapt with continued benefits for systems after a defined period of time.
Implementation determinants	Delivery of the intervention	Delivery of the implementation strategy
1. Context	Aspects of the larger social, political, and economic environment that may influence intervention implementation [41].	Aspects of the larger social, political, and economic environment that may influence delivery of the implementation strategies
2. Acceptability	Perceptions among the delivery team that a given intervention is agreeable, palatable, or satisfactory [16].	Perceptions among the support system that implementation strategies are agreeable, palatable, or satisfactory.
3. Adaptability	Extent to which an intervention can be adapted, tailored, refined, or reinvented to meet local needs [27].	Extent to which implementation strategies can be adapted, tailored, refined, or reinvented to meet the needs of organizations at scale-up.
4. Feasibility	Perceptions among the delivery team that an intervention can be successfully used or carried out within a given organization or setting [16].	Perceptions among the support system that implementation strategies can be successfully used or carried out at scale within different organizations or settings.
5. Compatibility (appropriateness)	Extent to which an intervention fits with the mission, priorities, and values of organizations or settings [17].	Extent to which implementation strategies fit with the mission, priorities, and values of organizations at scale-up.
6. Cost	Money spent on design, adaptation and implementation of an intervention [42].	Money spent on design, adaptation and delivery of implementation strategies.
7. Culture	Organizations’ norms, values, and basic assumptions around selected health outcomes [27].	Organizations’ norms, values, and basic assumptions around selected implementation strategies.
8. Dose (satisfaction)	Delivery team’s satisfaction with an intervention and with interactions with the support system [38].	Support system’s satisfaction with implementation strategies.
9. Complexity	Perceptions among the delivery team that a given intervention is relatively difficult to understand and use; number of different intervention components [27, 37].	Perceptions among the support system that implementation strategies are relatively difficult to understand and use; number of different strategies. Related to implementation setting.
10. Self-efficacy	Delivery team’s belief in its ability to execute courses of action to achieve implementation goals [43].	Support system’s belief in its ability to execute courses of action to achieve implementation goals.

*Note:* Indicators are defined as to whether they assess delivery of the intervention to participants (by delivery partners) OR to delivery of implementation strategies at the organizational level (by those that comprise a support system). Where similar terms were collapsed, the term preferred by the expert group is numbered while the synonymous term is bracketed. Several indicators were grouped because they had similar or shared definitions (dose delivered/dose; compatibility and appropriateness; sustainability and maintenance; dose/satisfaction). Four indicators were excluded from the tables based on the opinion of the expert group that participated in rounds 4 and 5: implementation, recruitment, efficacy (of interventions) and effectiveness (of interventions)

Implementation evaluation during scale-up often assesses both delivery of the intervention (at the participant level) and delivery of implementation strategies (at the organizational level). Therefore, the expert group considered whether indicators measured delivery of an intervention or delivery of an implementation strategy. Although indicator names did not change, level of measurement is reflected in nuanced definitions. This difference is illustrated in the second and third column of Table 3. For example, to assess delivery of the intervention, *dose* measures the amount of intervention delivered to participants by the providers/health intermediary (we refer to this as the delivery team). Whereas, *dose* for assessing delivery of implementation strategies refers to the amount or number of intended units of each implementation strategy delivered to health intermediaries by both scale-up delivery and support systems, at the organizational level (we refer to this collectively as the support system [31]). This reiterates the need to define indicators based on phase of trial along the continuum from feasibility toward scale-up (Fig. 1) and to consider implementation across levels of influence from providers most proximal to participants to those more distal within contexts where the intervention is delivered.

## Discussion

Since the launch of the Millennium Development Goals in 2000 [44], the health services sector has continued to build a foundation for scale-up of effective innovations. However, there is still a great need to evaluate implementation and scale-up of effective health promoting interventions. There are many possible reasons for the relatively few PA and nutrition implementation studies and the dearth of scale-up studies. The diverse quality and consistency of the few published reports that exist and finding a way through the maze of implementation and scale-up frameworks, indicators, and measures and tools are likely among them [1]. As there are sector-based differences in how terms are defined, and language is used, how users interpret and translate results is also not straight forward.

To address this deficit, we create a pathway for researchers who seek to differentiate between implementation at small and large scale and evaluate implementation across the program life cycle [17, 35, 45]. By identifying relevant frameworks and common indicators, and defining them in a standardized way we create an opportunity for cross-context comparisons, advancing implementation and scale-up science in PA and nutrition. Ideally, we would rely upon empirical evidence from large-scale intervention studies to construct a short list of implementation frameworks and process models, and a minimum data set of indicators. However, these data do not yet exist. Thus, we relied on those with experience in the field to share their knowledge and expertise. Finally, our intent was not to prescribe any one approach, but to suggest a starting place to guide evaluation for researchers who choose to implement and scale-up PA and nutrition interventions. From this starting place we envision that researchers will adapt, apply, and assess implementation approaches relevant to the context of their study.

### Theories and frameworks

Theories and frameworks serve as a guide to better understand mechanisms that promote or inhibit implementation of effective PA and nutrition interventions. We are by no means the first to seek clarity in classifying them. Within the health services sector, Nilsen [10] created a taxonomy to “distinguish between different categories of theories, models and frameworks in implementation science”. Approaches were couched within three overarching aims; those that describe or guide the process of implementation (process models), those that describe factors that influence implementation outcomes (determinant frameworks, classic theories, implementation theories) and those that can be used to guide evaluation (evaluation frameworks). Others collapsed theories and frameworks under the umbrella term models, and differentiated among broad, operational, implementation and dissemination models [9].

Thus, we extend this earlier work [9, 10], while seeking to further clarify terms for those conducting PA and nutrition research. Based on our results, we refer to both determinant frameworks and implementation theories as *frameworks* and reserve the term models for *process models* that have a more temporal sequence and guide the process of implementation. Notably, as most classification systems [10] do not distinguish between implementation and scale-up frameworks we added that categorical distinction (Table 1).

#### Implementation frameworks and process models

Classifying frameworks proves enormously challenging. Although differences often reflect sector based ‘cultures’ [9], we found that even researchers in the same general field define and use the same term quite differently. ‘Frameworks’ named by our study participants traversed the landscape from behaviour change theories to more functional process models. However, most were among the 61 research based models used in the health services, health promotion, and health care sectors [9]. Of these, most can be traced back to classic theories such as Rogers’ Diffusion of Innovations [37] and theories embedded within psychology, sociology, or organizational theory [10].

Differences reflect that implementation and scale-up research spans a broad and diverse range of disciplines and sectors, with little communication among groups [10]. Frameworks selected in our study might also denote geographic diversity of participants (6 countries represented) and implementation and scale-up research experience (3 to > 20 years). Settings where participants conducted their research also varied (e.g. community, health and school sectors) as did their focus on level of influence across a continuum from participants to policy makers, as per the socioecological model [46]. To achieve some clarity, our expert group differentiated among implementation and scale-up frameworks. Most implementation frameworks that assess delivery of an intervention at small scale can be used to describe and evaluate the process of delivering the intervention at broad scale. However, evaluation approaches at scale may be quite different and focus more so on ‘outer setting’ factors that influence scale-up [27]. Within scale-up, the expert group differentiated process models [33] from foundational or comprehensive dissemination theories or conceptual models [37].

Determinant frameworks depict factors associated with aspects of implementation [47]; they do not explicitly detail *processes* that lead to successful implementation [10]. Among determinant frameworks, the Framework for Effective Implementation [17] and CFIR [27] ranked most highly. Although participants did not provide specific reasons for their selection, both frameworks are flexible and identify critical factors that influence implementation along a continuum that spans policy and funding to aspects of the innovation (intervention). Framework for Effective Implementation [17] and CFIR [27] were generated from within different sectors (prevention and promotion versus health services, respectively) and use different terminology. However, there are many commonalities. Importantly, both were designed to be adapted to the local context to support implementation and scale-up.

Birken and colleagues [12] suggested that given myriad choices, implementation and scale-up frameworks are often selected in a haphazard fashion ‘circumscribed by convenience and exposure’. We argue that choice is likely more intentional, although the ‘best fit’ is not always clear for users. Interestingly, we noted a paradox as elements of preferred frameworks did not precisely align with the minimum data set of indicators deemed most relevant by these same participants (e.g. specific practices and staffing considerations) [17]. Currently, there is no supporting evidence that guides researchers to ‘preferred’ frameworks or clearly delineates indicators associated with framework constructs. A set of criteria to help researchers and practitioners select a framework (*and indicators*) may be preferable to more prescriptive guidelines [12]. This speaks to the need for discussion among sectors to clarify how frameworks might be adapted to setting and population and aligned with well-defined and measurable indicators.

#### Scale-up frameworks and process models

The most frequently noted classic theory, Rogers’ Diffusion of Innovations theory [37] identifies a diffusion curve and factors that influence adoption and implementation. Rogers also theorized about diffusion of innovations into organizations [37]. This seminal work influenced many other conceptual, implementation and scale-up frameworks. Among them is Greenhalgh et al.’s conceptual model for the spread and sustainability of innovations in service delivery and organization [26]. This comprehensive conceptual model highlights determinants of diffusion, dissemination, and sustainability of innovations in health service delivery organizations.

It became apparent that scale-up was much less familiar to participants. This is consistent with the literature as only 3% of PA studies were interventions delivered at scale [3]. When asked about scale-up frameworks participants instead cited four process models that could apply to most public health initiatives [30, 33–36]. Popular among them, WHO/Expand Net Framework for Action incorporates elements of the broader environment, the innovation, the user organization(s), and the resource team, juxtaposed against scale-up strategies [48]. Further, although there are different types of scale-up (e.g. *vertical* – institutionalization of the intervention through policy or legal action and *horizontal* – replication of the intervention in different locations or different populations) [48], participants did not differentiate among them. Results may reflect that participants were attuned with the process of operationalizing interventions at small or large scale more so than with concepts that guide the process and evaluation of scaling-up.

There are many common elements and steps across scale-up frameworks/models [30, 31]. These span attributes of the intervention being scaled-up to the broader socio-political context to how research and evaluation results are fed back to delivery partners and users to inform adapting the implementation process [1]. Although the origins of Yamey’s scale-up framework is in global health [32], it is accessible, practical and can be easily applied to PA and nutrition scale-up studies. Rather than distinct or prescriptive classification systems, others [12] recommend developing criteria to support researchers to select an appropriate framework. Within this rather ‘murky’ field, our findings provide a starting place for researchers who wish to scale-up their interventions and evaluate implementation and scale-up processes. There may be many other frameworks beyond what we highlight in this study to address specific implementation or scale-up research questions, contexts, settings, or populations.

### Creating a minimum data set of indicators

To create a ‘minimum data set’ of indicators for those in PA and nutrition research, we relied upon the work of Proctor et al. [16] and others [49, 50] who are advancing taxonomies for implementation outcomes. We are not the first to note that implementation science is rife with different and often inconsistent terminology [16, 51]. As implementation research is published in different disciplinary journals this may come as no surprise. We share the strong view of others [16, 52] that it is imperative to develop a common language for implementation indicators if we are to advance implementation research in our field. We offer the minimum data set as another step toward achieving a more standardized approach.

Rank order results appeared to reflect the scope of research conducted by participants. For example, the expert group more often conducted and evaluated implementation and scale-up studies in collaboration with government and policy makers. Thus, while *acceptability* was ranked number one by Delphi survey participants, *reach* was ranked number one by the expert group. Reach similarly surfaced as the dominant theme (considered the ‘heart of scalability’) by senior population health researchers and policy makers [53]. Also telling is the greater importance placed on sustainability (as an extension of scale-up) by the expert group.

Our efforts to differentiate implementation outcomes from determinants within implementation indicators has not been discussed at length previously. Within the health service sector, Proctor et al. [16] identified eight implementation outcomes (i.e., acceptability, adoption, appropriateness, costs, feasibility, fidelity penetration and sustainability). However, they were also referred to as “implementation factors” (e.g. feasibility) for implementation success. We differentiate between implementation determinants (e.g. satisfaction and acceptability) and implementation outcomes (the end result of implementation efforts; e.g. reach). However, an implementation indicator may serve a dual role [54] as either an outcome or a determinant depending on the research question. For example, it may be of interest to assess how acceptability (defined here as an implementation outcome variable) is influenced by perceived appropriateness, feasibility, and cost (defined here as implementation determinant variables) [16]. Conversely, it may be of interest to assess whether acceptability (as an implementation determinant variable) influences adoption or sustainability (implementation outcome variables) [16]. To our knowledge there is no formal taxonomy that describes whether an indicator is a determinant or an outcome.

Further, implementation indicators may be named, defined, and classified differently across sectors [51]. For example, ‘reach’ (public health) and ‘coverage’ (global health) both refer to the proportion of the intended audience (e.g., participants or organizations) who participate in or offer an intervention. To add further complexity to these issues, almost all implementation indicators serve as determinants of health outcomes in implementation and scale-up studies.

### Measures and tools

There is a great need to develop appropriate, pragmatic measures and tools that can be applied to implementation studies conducted in real world settings [16]. Currently for implementation evaluation, assessment spans quantitative (surveys) to qualitative (interviews/ focus groups) approaches. Many researchers devise “home-grown” tools and pay little attention to measurement rigour [16]. Lewis et al. [20] used a 6-domain, evidence-based assessment rating criteria to measure quality of quantitative instruments used to assess implementation outcomes in mental or behavioural health studies. Of 104 instruments, only one demonstrated at least minimal evidence for psychometric strength on all six criteria.

Measures and tools are currently being developed to assess different aspects of implementation and scale-up in the health promotion sector [55–57]. However, producing standardized, valid and reliable tools in a context-driven, dynamic science is a challenge. Indeed, it may not be feasible to re-establish validity and reliability when instruments are adapted to different contexts at scale-up, given the time demands of a real world environment, capacity and cost to do so.

### Strengths and limitations

Strengths of our study include our evidence informed process and the use of broader implementation science literature to guide how frameworks and indicators were represented and defined. Further, all participants had experience with implementation and/or scale-up evaluation. Finally, we included two in-depth, in-person follow up meetings with senior scientists to clarify findings and to address discrepancies.

Study limitations include the snowball sampling process we adopted to recruit participants beginning with researchers who were all affiliated with one known organization. We did not attempt to create an exhaustive list of those conducting studies in PA and nutrition; nor did we recruit implementation scientists or practitioners outside these topic areas. Thus, we may have excluded authors who assessed implementation of pilot and intervention studies or other eligible researchers not identified through our snowball sampling procedure. Second, as in any Delphi process, data were subjective and based on the availability, expertise, and knowledge of participants. Thus, recommendations were ranked based on what a limited number of experts considered “relevant and most frequently used” frameworks and indicators. To our knowledge there is no empirical evidence to verify among these, which frameworks or indicators are ‘best’ for evaluating implementation and scale-up of PA and nutrition interventions. Third, although an a priori objective was to link frameworks to indicators and indicators to measures and tools, few participants described any measures and tools. This perhaps reflects the state of measurement in the field overall. Fourth, given our focus on providing a roadmap for those in research and evaluation, we only included practitioners identified through our snowball sampling approach. However, we acknowledge that the process of scale-up could not be conducted without the support of key stakeholders. Finally, our findings apply to implementation and scale-up of PA and nutrition interventions; they may not be generalizable to other disciplines.

## Conclusions

Advancing the science of scale-up requires rigorous evaluation of such initiatives. The priority list of implementation frameworks and process models, and a ‘minimum data set’ of indicators we generated will enhance research planning and implementation evaluation in PA and nutrition studies, with a focus on studies proceeding to scale-up. Advancing our science is predicated upon increased efforts to develop adaptable measures and tools.

## Supplementary information

**Additional file 1.** Instructions to participants (for round 1 of the Delphi process).

**Additional file 2.** Interactive spreadsheet provided to participants in round 2 of the Delphi process.

## Data Availability

The datasets used in the current study are not publicly available as stipulated in our participant consent forms but are available from the corresponding author on reasonable request.

## Abbreviations

CFIR: Consolidated Framework for Implementation Research
ISBNPA: International Society of Behavioural Nutrition and Physical Activity
SIG: Special Interest Group
SIRC: Society for Implementation Research Collaboration
PA: Physical activity
WHO: World Health Organization
