# Supplementary material for: Implementation and scale-up of physical activity and behavioural nutrition interventions: an evaluation roadmap
Source: Int J Behav Nutr Phys Act. 2019 Nov 7;16:102. doi: 10.1186/s12966-019-0868-4 (PMC6839114; doi:10.1186/s12966-019-0868-4)
Supplement: Supplementary file 1 — Additional file 1. Instructions to participants (for round 1 of the Delphi process). [file 12966_2019_868_MOESM1_ESM.docx]

Supplementary file 1

Instructions to participants (at each round of the Delphi process)

**First round:**

1. Please answer the questions below so that we can learn a little more about you.

1. What is your age? _________________________

2. What is your gender? _________________________

3. What is your country of residence? _________________________

4. How many years of years of experience do you have conducting implementation and/or scale-up science research? _________________________

5. Do you self-identify as primarily an implementation scientist, scale-up scientist or both? _________________________

1. What is your email address? We will use this information for subsequent contacts and to link your responses across the study’s three “rounds” (time points).

Email address: _________________________

1. Below is a question and list of responses (items) generated by participants of an ISBNPA workshop. Participants have answered this question in terms of how it relates to **implementation science**. You will see a separate list for scale-up science on the next screen.

Please take a look at the generated items and with only **implementation science in mind**:

1. Add any items that you feel are missing.
2. Identify any items that you feel should be excluded.
3. If you feel that any of the items above are misclassified (i.e., misgrouped), please identify the item(s) below and suggest a different classification (e.g., “*Item_name* is not a *framework*. This item should be grouped under *model* instead”). If you feel an item has been misclassified as related to implementation science instead of scale-up science (only), please let us know here as well (e.g., “*Item_name* should be grouped under scale-up science, not implementation science”).

**Question**

*“What are the: a) models, frameworks, and theories; and b) indicators, measures, and tools that you believe are important in the realm of Implementation Science as it relates to physical activity and nutrition?”*

**Initial responses (items)**

1. Below is a question and list of responses (items) generated by participants of an ISBNPA workshop. Participants have answered this question in terms of how it relates to **scale-up science**.

Please take a look at the generated items and with only **scale-up science in mind**:

1. Add any items that you feel are missing.
2. Identify any items that you feel should be excluded.
3. If you feel that any of the items above are misclassified (i.e., misgrouped), please identify the item(s) below and suggest a different classification (e.g., “*Item_name* is not a *framework*. This item should be grouped under *model* instead”). If you feel an item has been misclassified as related to scale-up science instead of implementation science (only), please let us know here as well (e.g., “*Item_name* should be grouped under implementation science, not scale-up science”).

**Question**

*“What are the: a) models, frameworks, and theories; and b) indicators, measures, and tools that you believe are important in the realm of Scale-Up Science as it relates to physical activity and nutrition?”*
